# Epidemiology and Screening of Developmental Dysplasia of the Hip in Europe: A Scoping Review

**DOI:** 10.3390/reports7010010

**Published:** 2024-02-01

**Authors:** Emmanuela Dionysia Laskaratou, Anna Eleftheriades, Ioannis Sperelakis, Nikolaos Trygonis, Periklis Panagopoulos, Theodoros H. Tosounidis, Rozalia Dimitriou

**Affiliations:** 1 Department of Orthopaedics and Traumatology, University Hospital of Heraklion, University of Crete, 715 00 Heraklion, Greece; emmalsk@hotmail.gr (E.D.L.); ysperelakis@yahoo.gr (I.S.); nickos.tr@hotmail.com (N.T.); ttosounidis@yahoo.com (T.H.T.); 2 Faculty of Medicine, University of Basel, 4056 Basel, Switzerland; a.eleftheriadi@unibas.ch; 3 3rd Department of Obstetrics and Gynaecology, Attikon University Hospital, National and Kapodistrian University of Athens, 124 62 Athens, Greece; paninosrafaela@yahoo.gr

**Keywords:** developmental hip dysplasia, neonatal screening, Europe

## Abstract

Developmental hip dysplasia or developmental dysplasia of the hip (DDH) includes a wide range of deformities of the hip, such as congenital dysplasia, subluxation, and dislocation. It is usually identified through neonatal screening during the first 6–8 weeks of life. The incidence of DDH ranges from 1–7% in neonates among some populations, but this may vary among different ethnicities and countries. A consensus about the ideal age for screening has not been reached to date. The aim of this study is to summarize the existing data regarding the incidence of congenital hip dysplasia and screening tests among European countries. The authors conducted a systematic search in PubMed/Medline and Scopus and collected original studies published in English, French or German. The incidence of DDH presents fluctuations, not only among European countries, but also within the same country. There is no unanimity regarding the screening methods of DDH; in some countries, universal ultrasound is proposed as the basic screening method for neonates for DDH; in other countries screening is performed only in high-risk cases. More robust data are needed to conclude which screening approach is associated with improved long-term outcomes.

## 1. Introduction

Developmental dysplasia of the hip (DDH) describes a spectrum of conditions associated with the development of the hip in neonates and children. Laxity of the hip and immaturity of the acetabulum during the first few weeks of life is a normal finding; the laxity resolves, and the acetabulum develops normally. DDH encompasses numerous persisting alterations of the hip joint, ranging from dysplasia to real dislocation of the hip. The manifestations of DDH are variable; the femoral head can be within the acetabulum (enlocated), partially displaced (subluxated), or fully displaced from the acetabulum (dislocated) [1].

The differential diagnosis of DDH includes teratologic and neuromuscular hip dysplasia as well as other conditions such as proximal femoral deficiency [2]. Typical DDH generally refers to otherwise healthy infants; however, hip dysplasia and instability may be related to other conditions. Teratologic hip dysplasia refers to hip dysplasia occurring in association with various conditions or syndromes such as arthrogryposis, etc. Neuromuscular hip dysplasia refers to hip instability and dysplasia occurring in association with conditions such as spina bifida or cerebral palsy, characterized by weakness or spasticity in some of the hip girdle muscles. A careful review of the infant’s medical and family history helps to exclude other congenital or neuromuscular causes of hip instability, which is very important since the management of the different forms of dysplasia in infants differs significantly [2,3]. Important risk factors for DDH include female sex, family history of DDH, tight lower extremity swaddling, and breech presentation at ≥34 weeks of gestation regardless of the mode of delivery or a successful external cephalic version [4]. Torticollis, plagiocephaly, metatarsus adductus, clubfoot, being the firstborn, oligohydramnios, birthweight > 4 kg, and multiple gestation pregnancy are also thought to be risk factors even though there is a lack of evidence to support these associations [5]. Overall, breech presentation appears to be the most important risk factor, with the incidence of DDH reported up to 27% [6].

Timely diagnosis through the assessment of risk factors, physical examination, and the correct use of imaging techniques can lead to appropriate early treatment and therefore to the prevention of certain complications of DDH long-term sequelae, such as dislocation of the hip, avascular necrosis of the femoral head, and degenerative osteoarthritis. To date, a consensus regarding the ideal approach for screening has not been reached [3].

Across the globe and particularly in Europe, there are different screening programs at national level. This can be explained by the lack of universal instructions regarding the screening methods for DDH. Screening recommendations for DDH vary significantly from country to country. Some bodies recommend screening of all infants, whereas others recommend screening of high-risk children [7].

The age at which screening is performed as well as the screening approach (clinical examination, ultrasonography, risk stratification) also vary between different countries. For example, in the German-speaking countries and Italy, screening with ultrasound is performed at an early age, usually at 6–8 weeks of life. Of note, in German-speaking countries, Graf’s practice is part of new-born screening for DDH. In more detail, the hip is evaluated by measuring two angles, the alpha angle (α) and the beta (β) angle. They are made by three lines, drawn from the acetabular lateral edge, the bottom of the acetabulum, and the acetabular labrum. These lines have to be noted down in order to determine the bony roof angle (known as the α angle) and the cartilage roof angle (known as the β angle). In a centered hip, an angle is normally more than 60°, and the beta angle less than 55° [1]. In The Netherlands, screening with ultrasound is recommended for infants at the age of three months when risk factors have been reported, or earlier if clinical instability of the hips is ascertained during physical examination, which is performed at one week, one month, and three months of age. Generally, it is preferable not to perform ultrasound before six weeks of life unless there is clinical instability in the hips, due to the laxity of neonatal hips, which resolves by six weeks of age [3,4]. The literature is limited regarding the epidemiology of DDH in certain parts of Europe, particularly in Greece, the Balkans, as well as in eastern and southern Europe. It is reasonable to assume that the lack of evidence, partially, stems from the discrepancies between national screening programs and relevant diagnostic criteria and tools [7]. 

In the absence of consensus regarding screening programs for DDH among European countries, a systematic review summarizing the existing evidence and highlighting the knowledge and practice gaps in the field is relevant. Commissions consisting of orthopedics, pediatricians, and obstetricians from different European countries could benefit from such results to design guidelines for DDH screening programs for Europe. The objectives of our study were to summarize the existing knowledge regarding the epidemiology and the screening programs in the region of Europe and indicate relevant knowledge gaps. 

## 2. Materials and Methods

**Figure 1:**
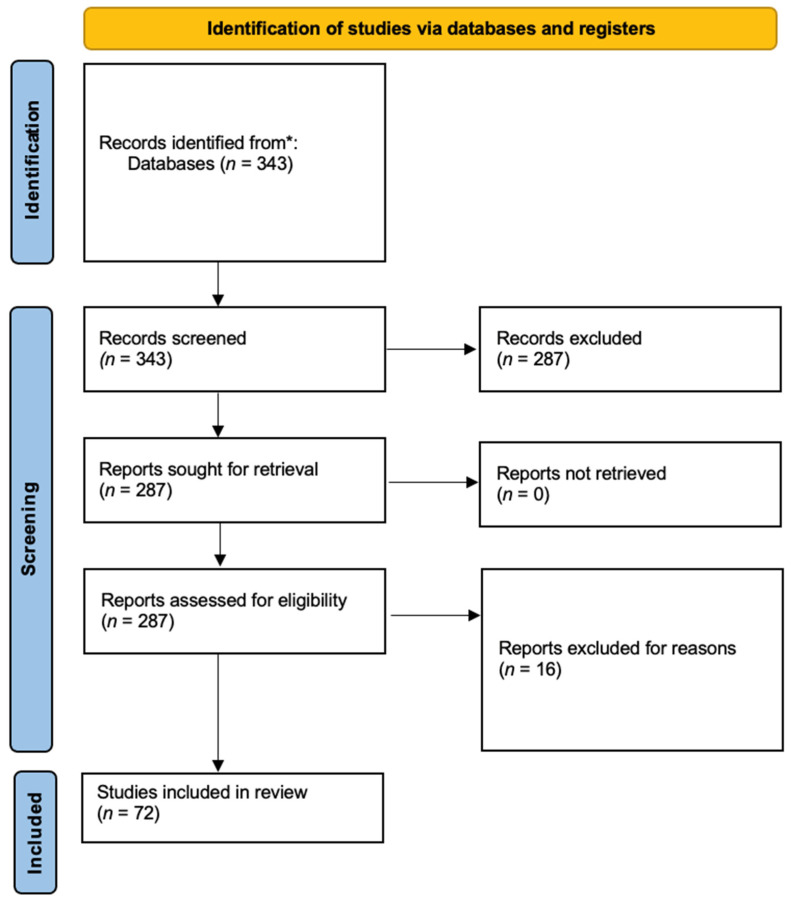
Flow diagram showing the numbers of titles and abstracts identified and screened and the full-text research papers assessed for eligibility and included in the qualitative synthesis. The included databases were (*) PubMed/Medline, Embase, the Cochrane Library/Cochrane Central Register of Controlled Trials (CENTRAL).

To identify relevant peer-reviewed publications and gray literature, the authors searched PubMed/Medline, Embase, and the Cochrane Library/Cochrane Central Register of Controlled Trials (CENTRAL) up until 15 October 2022. The reference lists of the selected sources and relevant systematic reviews were also manually searched to identify potentially relevant resources. The search terms “Developmental Dysplasia of the Hip” [MeSH], “Screening” [MeSH], “epidemiology [Subheading]”, “Europe” [MeSH], and “Europe Eastern” [MeSH] were used in combination with Boolean operators (AND, OR), when appropriate. Studies were included if they fulfilled all of the following eligibility criteria: (1) ongoing or published clinical studies reporting on DDH epidemiology and/or screening methods and programs in the WHO Europe region and/or the European Union, and (2) epidemiological analyses and reports. A study was excluded if it met at least one of the following criteria: (1) non-English, French, or German publication language in order to be more familiar with the used language (members of the research team can speak all three languages), (2) opinion article, perspective, or letter to the editor, (3) focuses on different region(s), and (4) no “Congenital Dislocation of the Hip” was used in the search terms, since it is an older definition. No sample size restriction was applied when screening for eligible studies. Disputes in the selection of relevant studies were discussed between the two primary authors and a senior author until a consensus was reached. The literature was searched and reported according to the Preferred Reporting Items for Systematic Reviews and Meta-Analysis (PRISMA) extension for Scoping Reviews (PRISMASc) (Figure 1) [8]. 

## 3. Results

During the initial review of the literature, 343 studies were identified, and 271 studies were excluded. From the remaining ones, 26 concerned the epidemiology of DDH, 21 focused on DDH screening and 25 concerned both the epidemiological and screening aspects of DDH in the European region. The oldest study was conducted back in 1964, and the most recent was conducted in 2020. A detailed overview of the included studies and their key outcomes is presented in Table 1.

### 3.1. Epidemiological Studies

The incidence of DDH in Europe ranged from 0.59 per 1000 live births to 27.53 per 1000 live births, which was the maximum limit of incidence of DDH in Europe, observed in Hungary. Furthermore, incidence also ranged significantly in Greece, especially in Crete, as it was reported to be 10.83 per 1000 live births [9,10,11]. Differences in the rates of DDH can be observed not only among different countries in Europe, but also between different areas in the same country; for example, the incidence of DDH diagnosed in newborns varied between three hospitals in Northern Sweeden. The incidence was 10.0, 7.1, and 3.5 per 1000 live births in different hospitals [12]. Unfortunately, very few epidemiological studies have examined the incidence of DDH, and due to the insufficient data, we cannot draw general conclusions regarding the incidence of DDH in Europe [7]. 

The effects of certain risk factors were also studied regarding their association with the development of DDH, namely, multiple pregnancies, increased gestational age, birthweight, and experience/competence of the physician in performing the neonatal screening tests for DDH. Rühmann et al. observed a correlation between heredity and breech presentation and the need for open procedures for DDH [13].

Rosendahl et al. concluded that breech presentation during birth appeared to be a significant risk factor affecting females only and having a sibling or a parent with DDH was a more significant risk factor for the appearance of DDH than having a second- or third-degree relative with DDH [14].

### 3.2. DDH Screening

There is no consensus regarding the appropriate screening methods for DDH in Europe or the age at which screening should be performed. Most studies recommend sonographic and/or clinical assessment as a screening tool. Krikler et al. conducted a comparative study at the Royal Orthopedic Hospital in the UK and concluded that screening can be effective, provided that all newborns are screened at birth and cases with risk factors are followed up by a trained team with an appropriate follow-up protocol and supervised by an orthopedic surgeon [15]. A study from Norway that aimed to examine the impact of adding an ultrasound examination to the screening strategy showed that the treatment rate was doubled (non-operative) without influencing the already low numbers of late-diagnosed cases [16]. Laborie et al. conducted a randomized trial regarding the use of universal ultrasound; ultrasound use increased the early diagnosis of DDH to a non-statistically significant level; however, in the long-term this was not clinically significant as it did not influence the frequency of chronic complications of the disease [17]. A study by Čustović et al. also appears to be in favor of a simple clinical examination; it was shown that unilateral limited hip abduction could a valuable clinical sign of DDH (positive predictive value = 40.3% and negative predictive value = 80.4%), which could be used for diagnostic purposes [18]

There has been, however, contradictory evidence regarding the effectiveness of DDH screening with a simple clinical examination, as many cases of DDH still remain undiagnosed when using this approach [7]. Reidy et al. investigated the screening effectiveness of the hip examination at 6–8 weeks, performed by a general practitioner, and found that the sensitivity of examination was only 19.4%, as well as that four of five children with DDH remained undiagnosed [19]. Two studies from Germany and one from Wales concluded that the universal use of ultrasound for screening purposes reduced the rate of open surgery for DDH [20,21,22]. Günther et al. also confirmed the effectiveness of universal ultrasound screening for DDH in Germany [23]. 

Engesaeter et al. pointed out that only a small percentage, 8% of those who underwent total hip arthroplasty due to hip dysplasia, were reported to have it at birth, concluding that clinical testing for neonatal hip dysplasia is insufficient by itself as a screening method for dysplastic hips that require total hip arthroplasty in young adulthood [24]. In this context, Elbourne et al. recruited infants with clinical hip instability from 33 centers in the UK and Ireland and randomized them to undergo either ultrasonographic hip examination or clinical assessment alone. They showed that the use of ultrasonography reduced abduction splinting rates and was not associated with higher rates of surgical treatment by 2 years of age or significantly higher health-service costs [25].

The use of ultrasound in selected groups has also been proposed. Salut et al. suggested the use of ultrasound for a high-risk group, namely 30-day-old female neonates [26]. However, ultrasonography can also present certain limitations; Muresan et al. found that the most frequent stage of DDH detected through ultrasound was type IA, and the rarest stage was III. The incidence of hip dysplasia stage III diagnosed through ultrasound examinations in the central region of Romania was 0.2% [27].

As far as radiography is concerned, it seems not to be preferred as a screening tool. Nevertheless, it could be used in the context of screening at four months of age for babies at increased risk of DDH who had been normal at birth [28]. Wenger et al. examined the radiographic outcomes at 1 year of life in newborns undergoing early treatment for neonatal hip instability. It was found that even in newborns who are diagnosed and treated, the radiographic differences may remain after 1 year of life [29].

The above results reflect the reality that exists in Europe regarding DDH epidemiology and screening. The incidence of DDH in Europe presents fluctuations in different countries [7]. As mentioned above, the incidence of DDH in Europe ranged from 0.80 per 1000 live births to 27.53 per 1000 live births. The incidence of late diagnosis was low, probably due to raised awareness regarding the timely diagnosis of DDH. For instance, the incidence of late diagnosis was 1.28 per 1000 live births in Southampton, during the period 1990–2016 [30]. 

**Table 1:** Studies concerning the epidemiology and screening of DDH that were included in the systematic review.

References	Focus of the Study	Country or Region	Time Period	Study Type	Objective	Key Outcomes
Perry DC, 1 November 2011 [31]	Association between clubfoot and DDH	UK	2002–2008	observational cohort	epidemiology	Among children with clubfoot and DDH, 5.9% will require treatment
Price et al., 1 June 2013 [32]	Evaluation of current screening practices; only two examinations: at birth and then at six to ten weeks of age	UK, Nottinghmam	1990–2005	retrospective review	screening	Established a final examination for DDH during the first six to nine months of life that would potentially prevent a significant increase in the presentation of DDH beyond walking age
Phelan et al., 1 June 2015 [33]	Incidence and treatment outcomes of DDH	Southest Ireland	2009	retrospective study	epidemiology	The incidence of DDH was estimated 6.73 per 1000 live births. The rate of open procedures was 1.08 per 1000 live births
Olsen et al., 1 February 2018 [16]	Evaluation of screening effectiveness when adding universal ultrasound hip examinations within three days of life	Norway, Kongsberg Hospital	1998–2006		screening	The treatment rate was doubled, without influencing the already low numbers of late cases
O’Grady MJ, 1 June 2010 [34]	Current practices in Ireland; most units (84%) were dependent on radiographs at 4–6 months for imaging hips; only two units primarily used ultrasound (10.5%)	Ireland	2006	prospective and retrospective study	screening	Selective ultrasound and examination by an experienced clinician are not widely practiced
Talbot CL, 1 September 2013 [35]	Evaluation of current screening methods; the Newborn and Infant Physical Examination (NIPE) programme in the UK recommends selective ultrasound screening for at-risk infants (breech presentation and family history)	UK	2008–2013	observational, longitudinal cohort study	screening	The risk of DDH in males referred with risk factors but clinically stable hips was found to be low
Wenger D, 1 December 2020 [29]	Evaluation of the addition of secondary screening for hip dislocations	Sweeden	2000–2009	retrospective analysis	screening	Secondary screening at 6–8 weeks, 6 months, and 10–12 months of age has decreased the age of late diagnosis in half of children that were not diagnosed through primary screening
Biedermann R, 1 October 2018 [36]	Incidence study	Austria	1998–2014	prospective follow-up	epidemiology	Incidence of DDH: 8 per 1000 live births, treatment rate: 1%
Geertsema D, 1 August 2019 [37]	Does delayed radiological hip screening at five months (versus ultrasound at 3 months) result in a higher incidence of persistent developmental dysplasia of the hip (DDH) at 18 months?	Northern Ireland	2011–2017	a prospective observational study	screening	No significant difference in incidence or severity of persistent DDH at 18 months between the two screening groups
Treiber M, 1 January 2008 [38]	To assess the results of the general screening program of newborns’ hips in Maribor between 1997 and 2005 in comparison to results from 1985 for the same region	Maribor	1997–2005	retrospective analysis	screening	Universal US in neonates led to a reduction in the overall treatment rate
Milligan DJ, 1 April 2020 [39]	To assess the quality of services for DDH in Northern Ireland (neonates with a positive screening examination should undergo ultrasound scanning)	Musgrave Park Hospital, Belfast, UK		prospective observational study	screening	Conformity to the regional neonate hip screening protocols led to reduction in the rates of open procedures and in the number of pelvic x-rays in infants
Maxwell Sl, 27 April 2002 [9]	Evaluation of screening methods in Northern Ireland	Northern Ireland	1983–1987	comparative retrospective study	epidemiology	The true incidence of DDH still remains unknown
Salut C, 1 November 2011 [26]	To identify the value of US screening for DDH. Systematic US evaluation of the hips using the Couture technique was performed at 1 month with all girls with a normal physical examination at birth over a 1-year period.	Limoges, France	2009	retrospective study	screening	A total of 74 abnormal hips undetected during the initial clinical evaluation in girls without risk factors were detected and treated
von Kries R, 1 February 2012 [20]	To assess the effectiveness of general ultrasound screening to prevent first operative procedures of the hip	Munich, Germany	1996–2001	case-control study	screening	Universal ultrasound reduces the rate of operative procedures for DDH
Barr LV, 1 January 2013 [5]	Do multiple births lead to a higher incidence of DDH and is there a need for selective US?	Hospital, Hills Road, Cambridge, UK	2004–2008	retrrospective study	epidemiology	Multiple births were not found to be a risk factor for DDH
von Kries R, 6 December 2003 [21]	Evaluation of current screening practices (universal ultrasound screening at first 6 weeks of life)	Germany	1996–2001	retrospective study	screening	US seems to prevent the many cases that require open surgery for DDH
Reidy M, 1 October 2019 [19]	Evaluation of current screening practices (neonate hip examination at 6–8 weeks of life)	UK	2006–2011	longitudinal observational study	screening	Four out of five children with DDH were not identified at 6–8 weeks. This screening method in its current form is not reliable
Jashi R, 12 May 2017 [40]	What is the prevalence of hip dysplasia among relatives with family history of hip dysplasia operated with periacetabular osteotomy (PAO)?	Denmark	1998–2014	cross sectional study	epidemiology	Females seemed to have an increased familial prevalence of hip dysplasia in comparison to males, but the increased prevalence was not statistically significant
McAllister et al., 1 November 2018 [41]	To calculate the risk of open surgery for DDH for infants up to 3 years old before and after the improved DDH detection services (Ortolani and Barlow tests shortly after birth vs. increased use of US)	Scotland	1997/98–2010/11	retrospective cohort study	epidemiology	The improved DDH detection services have reduced the number operative procedures for DDH from April 2005 and after
Davies R, 1 April 2020 [42]	Evaluation of current screening methods (clinical examination of the hips at 6–8 weeks)	Royal Blackburn Hospital, England	1996–2010	observational cohort	screening	6- to 8-week clinical hip assessments yielded sensitivity, specificity, positive predictive, and negative predictive values of 16.7%, 99.8%, 3.5%, and 100.0%, respectively
Colta RC, 1 January 2016 [43]	Analysis of risk factors for DDH	Bucharest, Romania	2013–2015	retrospective study	epidemiology	Increased gestational age and increased birthweight were associated with a higher risk for DDH
Laborie Lb., 1 September 2013 [17]	Evaluation of the addition of universal or selective ultrasound screening for DDH	Norway	2011–2013	randomized control trial	screening	Selective and universal ultrasound screening led to a nonsignificant reduction in the rate of late diagnoses
Muresan S,1 January 2019 [27]	Epidemiological study in Romania	Romania	2016	retrospective study	screening epidemiology	The most frequently diagnosed stage was type IA, and the rarest stage was III. The incidence of type III hip dysplasia was 0.2%
Peterlein CD, 1 June 2014 [44]	Epidemiological study in Marburg	Franziskus Hospital, Marburg	1985–2009	retrospective study	epidemiology	Breech presentation was significantly correlated with decentering and eccentric hips. Treatment of hip type II a according to Graf was inconsistent over time. Inexperienced physicians recommended therapeutic interventions more frequently
Broadhurst et al., 1 March 2019 [30]	Epidemiology study	Southampton Children’s Hospital, UK	1990–2016	observational study	epidemiology	The incidence of the late DDH diagnosis was 1.28 per 1000 live births and 71.1% of cases were detected in children between one and two years of age, with a female-to-male ratio of 4.2:1
Elbourne D, 21 December 2002 [25]	To assess the effectiveness and net cost of US compared with clinical examination alone	UK	1994–1998	randomised control trial	other	Utilizing ultrasound for infants with clinically detected hip instability lowered the need for abduction splinting
Lambeek AF et al., 1 April 2013 [4]	To assess the effect of a successful external cephalic version on the incidence of DDH requiring treatment in singleton breech presentation at term	The Netherlands	2006–2009	observational cohort study	epidemiology	Achieving a successful external cephalic version is linked to a reduced occurrence of DDH, but a notable proportion of children born following a successful external cephalic version still exhibit DDH
Kamath S et al., 1 May 2007 [45]	To determine if employing ultrasound for screening infants with risk factors has resulted in a decrease in late DDH	Whiston Hospital, UK	1992–2001		epidemiology	The annual incidence from 1992 to 1996 was reported to be 0.84, and from 1997 to 2001, it was 0.57 per 1000 births. The decline was not significant
Laborie et al., 1 April 2014 [17]	To assess the use of selective US as part of the screening programme	Bergen, Norway	1991–2006	prospective survey	screening epidemiology	Selective and universal ultrasound screening led to a nonsignificant reduction in the rate of late diagnosis in comparison to clinical examination
Čustović S et al., 1 August 2018 [18]	What is the relationship between the clinical sign of limited abduction of the hips and DDH?	Tuzla, Sweeden	2011–2012	prevalence study	screening epidemiology	Limited abduction of the hips had a positive predictive value 40.3% and a negative predictive value of 80.4% for DDH. Limitation of the abduction of the hip is an important sign of DDH
Giannakopoulou C, 1 January 2002 [11]	Epidemiology of DDH in Crete	Crete, Greece	1996–2000	retrospective study	epidemiology	The incidence of DDH was estimated to be 10.83 per 1000, higher than in the rest of Greece. Medical and family history and clinical examination contribute to the diagnosis of hip instability
Burger BJ, 22 December 1990 [46]	Epidemiology of DDH in The Netherlands	The Netherlands	1971–1979	prospective follow-up study	screening epidemiology	The percentage of missed dislocations of hips during screening was 0.02%. Dysplasia was detected at 5 months in 15% of infants with a positive family history and a negative Barlow test
Wenger D, 1 October 2013 [29]	X-ray findings at 1 year of age in children who received early treatment for neonatal instability of the hips		2002–2007	cohort study	screening epidemiology	The incidence of instability of hips in newborns was 7 per 1000 live births, and the referral rate was 15 per 1000. Girls were 82% of cases
Lewis K et al., 1 November 1999 [22]	What is the use of static US in DDH screening?	Morison Hospital, Wales	1988–1992	prospective study	screening	Simple static ultrasound is an effective screening test for DDH that should be applied to the general population
Lange AE, 16 March 2017 [47]	Compare the incidence of DDH between preterm and full-term babies	Germany	2002–2008	retrospective study	epidemiology	Preterm infants with gestational age < 36 weeks have a decreased risk of DDH
Rühmann O et al., 1999 [13]	The contribution of risk factors to the development of DDH	Germany	1987–1995	retrospective study	epidemiology	A higher rate of treatment needed was associated with family history of DDH, breech presentation, and female sex
Paton RW et al. 2005 [48]	The contribution of risk factors in screening with US	England, UK	1992–2002	prospective study	other	Risk factors and/or clinical instability of the hip was present in 7.4% of newborns, and 31% of the newborns with clinical instability had an associated risk factor. Family history, breech presentation, and foot deformity were the principal risk factors
Rosendahl K et al., 2 September 1996 [14]	Estimation of prevalence based on US diagnosis	Bergen, Norway	1988–1990	retrospective study	epidemiology	More females than males had minor and major dysplasia. Having a first-degree relative with DDH was found to be a risk factor. Breech presentation at birth was an important risk factor only for females
Jones D, 1 August 1977 [49]	Evaluation of current screening methods	England, UK	1968–1972		screening	Clinical examination of neonate hips is not an adequate screening tool; repeated examinations should be performed
Czeizel A et al., 1 November 1974 [10]	Incidence of DDH in Hungary	Budapest, Hungary	1970–1972	retrospective study	epidemiology	The incidence was estimated to be 28–71 per 1000 live births
Dunn PM et al., 1 May 1985 [50]	Comparison between the frequency of early and late diagnosis	Bristol, United Kingdom	1970–1979	retrospective study	epidemiology	The frequency of DDH diagnosis among neonates was 1.9%. The frequency of DDH diagnosis after the neonatal period ranged from 0.04 to 0.1%
Merk H et al., 1 October 1999 [51]	Evaluation of diagnostic strategy for DDH (clinical and sonographic screening examination)	Germany	1984–1995	retrospective study	epidemiology	Among the 4177 observed newborns, 39 cases of congenital dislocation of the hip joint in 27 children were found. After 12 months, a complete healing rate of 95 percent was exhibited with the functional management strategy
Zenios M et al., 1 October 2000 [52]	Impact of selective US screening on the late diagnosis of DDH in the Salford region	Hope Hospital, UK	1991–1995	retrospective study	screening	The incidence of late diagnosis was not reduced when compared with two previous cohorts at the same center
Sionek A et al., 1 March 2018 [53]	Effect of gender on the development of DDH	Warsaw, Poland	2008	retrospective study	screening	Gender seems to be a significant risk factor. Type IIa hips were more common in females
Boere-Boonekamp MM, 1 February 1998 [54]	To assess the validity of a standardized screening protocol for DDH in neonates (physical examination and possible referal)	Hengelo, The Netherlands	1992–1993	prospective studies	screening	The validity of this screening protocol for DDH is low. The addition of US to current screening protocols needs further evaluation
Krismer M et al., 1 January 1996 [55]	The contribution of US screening and treatment with a Pavlik harness to the incidence of DDH.	Austria	1979–1988	prospective studies	screening epidemiology	A significantly decreased dislocation rate was detected, suggesting that early use of US is valuable in the early detection of hip dislocation
Hindrraker et al., 1994 [56]	The contribution of intra-uterine factors to the hip instability of neonates (NIH)	Norway	1970–1988	regression analysis	epidemiology	The prevalence of NHI at birth was 0.9%: (0.6% in boys, 1.4% in girls). Among children born in breech presentation, the rate was 4.4%
Lennox IA et al., 1 January 1993 [57]	Evaluation of screening methods for DDH	Scotland	1980–1989	retrospective study	screening	Many dislocations have not been detected during newborn examinations (this referred to 0.13% of live births); 0.08% of live births required operative treatment
Falliner et al., 1998 [58]	Diagnosis and therapeutic management of DDH during the last seven years	Germany	1991–1997	retrospective study	Screening	Approximately 81% of newborns with clinical hip instability during birth obtained a sonographic DDH diagnosis. In 38% of them, it was possible to diagnose DDH within the first week of life
Engesaeter IØ et al., 1 June 2008 [24]	Does neonate hip instability increase the risk of total hip replacement (THR) in young adulthood?		1967–2004	retrospective study	other	Hip instability of neonates increases the risk of THR in young adulthood, although only 8% of those who had a THR due to hip dysplasia had unstable hips as neoantes. The authors conclude that clinical examination alone is insufficient as a screening method
Günther KP et al., 1 November 1998 [23]	Evaluation of universal ultrasound screening for DDH in Germany since 1996	Germany	05/1997–10/1998	cross-sectional study	screening epidemiology	US was performed too late and not at all in 35% of cases
Kramer et al., 1 January 1988 [59]	Evaluation of risk factors; does a positive family history contribute to DDH development?	Norway	1964–1984	comparative study	epidemiology	Having a first-degree relative with DDH resulted in a 10-fold increase in the DDH risk on average
Kramer et al., 1 January 1987 [60]	Evaluation of screening methods (with Barlow and Ortolani tests)	Norway	1964–1983	retrospective study	epidemiology	Breech presentation and early parity situation were linked to a higher risk of DDH. Neonatal screening programs in Norway may have demonstrated limited accuracy
Garvey M et al., 1 September 1992 [28]	Implementation of a screening programme for infants at four months of age who were clinically normal at neonatal examination but were considered to be ‘at risk’ for congenital dislocation (family history, breech presentation, persistent click)	Dublin	1986–1988	prospective pilot study	screening	Performing a hip radiography four months after birth significantly enhances neonatal screening for infants who face a higher risk of hip-related issues
Heikkilä E, 1 April 1984 [61]	Epidemiology of DDH in Finland	Uusima, Finland	1966–1975		epidemiology	The incidence was calculated to be 0.68% of liveborns
Husum et al., February 2019 [62]	Assessment of the pubo-femoral distance (PFD) in the lateral position as an indicator for unstable DDH	Aarhus University Hospital, Denmark	2013–2016		epidemiology	A PFD value above 4.4 mm was 100% sensitive and 93% specific for the detection of unstable DDH
Krikler SJ, 1 September 1992 [15]	Evaluation of screening methods; only clinical tests on new-born infants	Royal Orthopaedic Hospital, Birmingham|England	1980–1990	comparative study	screening	Screening for all neonates at birth and follow-ups of children at high risk for DDH by an appropriate and experienced team with a well-designed protocol are recommended
Godward et al., 18 April 1998 [63]	Evaluation of screening methods in the UK (universal clinical screening)	England	1993–1994	retrospective study	epidemiology	The percentage of neonates requiring an operative procedure for congenital dislocation of the hip in the UK was similar to that reported before screening was introduced
Ferris et al., 1 April 1997 [64]	Evaluation of protective factors against DDH	Ireland	1983–1995	retrospective study	other	Neonates in the supine or lateral position and the contribution of hip screening were correlated to a reduction in the incidence of late diagnoses of CDH. X-rays should be performed at 6 months old for infants with risk factors
Czeizel et al., 1 June 1975 [65]	Evaluation of risk factors (two family studies)	Hungary	1962–1967	retrospective study	epidemiology	The incidence of DDH was calculated 28.7 per 1000 live births. (In total, 523 infants required treatment from the total of 18,219 live births registered.) The impact of genetic predisposition could not be confirmed
Clausen et al., 1988 [66]	Epidemiological correlations between birth presentation, mode of delivery, and DDH	Denmark	1973–1986	retrospective observational study	epidemiology	Breech presentation was associated with DDH—13.3% of the neonates in this presentation were later diagnosed with DDH. No significant correlation between the mode of delivery and DDH diagnosis
Bernard et al., 1987 [67]	Evaluation of screening methods	West Midlands, England	1977–1983	retrospective observational study	screening	Repeated screening of high-risk newborns by physiotherapists effectively decreased the frequency of late DDH diagnoses
Bjerkreim et al., 1 May 1987 [68]	Late diagnosis of DDH in Norway during 1970–1974	Norway	1970–1974	retrospective observational study	epidemiology	Late DDH diagnosis may be associated with progressing dysplasia of the hip during the first twelve months of life
Macnnicol, 1990 [69]	Results of a 25-year screening program for neonatal hip instability	Edinburgh, Scotland	1962–1985	prospective study	screening	The frequency of late DDH diagnoses was 0.5 per 1000 live births when the examinations were carried out by junior physicians. Examination by or together with senior physicians is recommended to decrease the rates of late DDH diagnosis
Finlay et al., 1967 [70]	DDH epidemiology in Northern Ireland	Northern Ireland, United Kingdom	1962–1967	prospective observational study	epidemiology	The frequency of DDH diagnosis with the Barlow test was approximately 0.4% (60 cases among 14,594 infants)
Βeckman et al.,1977 [12]	DDH epidemiology in Nothern Sweeden	Sweden	1960–1973	prospective study	epidemiology	The incidence of neonatally diagnosed with congenital dislocation of the hip (CDH) ranged between 10.0, 7.1, and 3.5 per one thousand in different centers
Czeizel et al., 1972 [10]	DDH epidemiology in Hungary	Hungary	1962–1967	retrospective study	epidemiology	Among 108,966 infants, DDH incidence was 27.53 per 1000 live births; 72.4% of the diagnoses were made within the first 6 months of life
Konijnendijk et al., 2021 [71]	Evaluation of risk factors for DDH	The Netherlands	2021	cross-sectional study	epidemiology, risk factors	Breech presentation after gestational week 37.0 was associated with a more than threefold higher DDH risk
Guindani, 2021 [72]	Screening for DDH during the COVID-19 lockdown	Italy	2019–2020	retrospective study	screening	DDH-USS was the only screening in newborns during lockdown. The study observed a 22% decrease in DDH screening rates; 29% of DDH diagnoses were delayed, made at a mean age of 114 days
Koob et al., 2020 [73]	Epidemiology, risk factors	Central Europe	2013–2016	retrospective study	epidemiology, risk factors	Prematurity was inversely associated with DDH. Premature birth at gestational week 31 had the lower DDH incidence
Gunther et al., 1993 [74]	Epidemiology, risk factors	United Kingdom	1988–1990	retrospective study	epidemiology, risk factors	Multigravidae and similarly multiparous women had a statistically significantly reduced risk of having a baby with CDH. Babies born by Caesarean section or in breech position had an increased risk of CDH (statistically significant). Cases were more likely to have a family history of CDH than subjects who were screened but found to be normal

## 4. Discussion

In this systematic review, the authors examined the available literature regarding the incidence and screening of DDH, focusing on the European region. However, the current situation in other continents appears to be similar. 

There is no agreement regarding the optimal timing for ultrasound screening for DDH among European countries [7,75]. This results in a wide variation of screening practices in the European area, due to different healthcare systems as well as the difference in DDH incidence amongst European countries. Ultrasound screening is performed for all neonates in Italy, Germany, and Austria. On the other hand, in The Netherlands, ultrasound screening is selective and is performed at the age of three months in case of risk factors, or earlier in case of instability of the hips during the clinical examination performed at the age of one week, one month, or three months [76]. 

Kilsdonk et al. presented in summary different screening programs throughout Europe for the detection of DDH in neonates. It was found that a selective ultrasound screening program is performed in The Netherlands, Belgium, France, Sweden, Norway, Hungary, the United Kingdom, Ireland, and Portugal. Ultrasounds are conducted as early as one week of life, for example, in the Scandinavian countries, or later until four weeks of life, for example, in France [76]. In the UK, the NIPE guidelines recommend a clinical examination of the hip for all neonates, followed by a clinical examination at 6–8 weeks of life and referral for an US only in the case of risk factors [77].

A universal screening program is performed in Italy, Germany, Austria, Switzerland, Slovenia, and Slovakia. In Italy, an US is performed between four and twelve weeks of life, whereas in Germany and Slovenia, the timing depends on the presence of risk factors; in case of them, an US is performed as early as two weeks of life; otherwise, it is performed between four and five weeks of life. In Slovakia, an US is performed between one to twelve weeks of life [76]. In reviewing the existing literature, it can be assumed that the method of screening for DDH depends on neonatal age; for example, hips that are Barlow-positive at birth, may become stable in the first weeks of life, as the normal laxity of the hips due to maternal estrogens will be reducing [78].

The incidence of DDH in Europe seems to be much higher than in Africa and Israel and similar to that in Native Americans. In Africa, the incidence of DDH ranges from zero in Malawi to 0.15% in Ethiopia, generally lower than in Europe. A lower incidence may be attributed to the lack of diagnostic means or to limited access to healthcare facilities. Nevertheless, biological and environmental factors should also be considered; Graham et al. (2015) found out that DDH is rarely found among the Sub-Saharan African population [79]. Most mothers in Malawi back carry their babies during the first two years of life, in a position that is similar to that of the Pavlik harness. This could also contribute to the very low incidence of DDH in this country. Eidleman et al. conducted a 7-year prospective study (34,048 newborns were examined clinically and with US, 768 of whom were Ethiopian) and concluded that the incidence of DDH was 5.5% among the total Israeli population and 1.24% in Ethiopian Jews [80]. 

Τhe incidence of DDH was found to be lower in Asia than in Europe. Den et al. estimated the incidence of DDH in Japan at 0.076%. In Hong Kong, the prevalence of DDH was also at a low level: 0.87/1000 live births [81,82].

The incidence of DDH was found to be quite high in Native Americans, likely due to genetic factors. In Arizona Fort Apache Indians, the incidence was 31 per 1000 live births, probably due to endogamy and therefore a very limited variety of genes. In Fort Defiance, Arizona and Gallup, New Mexico, the incidence was estimated to be 67 per 1000 live births [83]. 

Certainly, the differences in DDH prevalence can also be attributed to a difference in diagnostic protocols across countries and continents. The American Academy of Pediatrics (AAP) and the DDH Task Force of Canada suggest clinical examination of newborns as a screening method for the detection of DDH mainly using the Ortolani test, and generally, the AAP does not recommend universal ultrasonographic screening [3,84]. Ultrasound is recommended for the ages of 6 weeks and 6 months for newborns with risk factors, including breech presentation, a positive family history, and female sex. The AAP suggests that most minor abnormalities of the hip, which are observed with ultrasonography at 6 weeks until 4 months, will retreat. The same approach regarding DDH screening is implemented in Asia. For example, in Hong Kong, universal physical examinations and selective ultrasounds are performed [82].

More awareness regarding DDH screening should be raised as it can lead to impaired functional outcomes in both children and adults. However, there is no direct proof that screening enhances functional outcomes, and the evidence supporting this is generally insufficient. According to expert opinion, early intervention is believed to be beneficial, although the evidence is mixed. Delayed diagnosis is associated with a higher likelihood of requiring surgical intervention and a higher rate of complications [7]. Despite the lack of strong evidence supporting its effectiveness in improving outcomes, universal screening for DDH is a well-established approach to addressing the disorder. However, the specific methods of screening vary significantly. Alongside physical examination techniques like the Barlow and Ortolani techniques and assessments of the hip abduction range of motion, static and dynamic ultrasound can be utilized to identify anatomical abnormalities and assess hip stability [7]. 

Some experts have suggested using risk stratification to guide the selective use of ultrasound in diagnosing DDH. It has been observed that females in breech positioning during delivery have the highest rate of clinical hip instability [1,2]. However, the effectiveness of ultrasound as a diagnostic tool for those with risk factors becomes less certain. Some healthcare systems have opted for universal ultrasound screening to reduce instances of late DDH diagnosis [7]. Utilizing ultrasound to further evaluate hips that exhibit instability during clinical examination may decrease the need for unnecessary treatment. However, it may also lead to a higher number of follow-up appointments for hips that ultimately normalize on their own. The reliability of ultrasound in classifying DDH is uncertain. There are potential drawbacks to screening for DDH, including the possibility of examiner-induced hip problems due to vigorous testing, increased risk of certain cancers from radiation exposure during follow-up radiographic tests, and parental psychosocial stress caused by the diagnosis and treatment [7]. However, none of these potential harms have been accurately quantified. 

To our knowledge, this is the second study that has systematically reviewed the epidemiology and screening of DDH, and the first that specifically refers to the situation in Europe. The adaptation of a broad search and reference-screening strategy are strengths of this literature review. The limitations include that a language criterion was applied, resulting in the exclusion of potentially relevant papers as well as a significant heterogeneity of the studies that were included in our review. Furthermore, no “Congenital Dislocation of the Hip” was used in the search terms, since it is an older definition and there is no other in use. This could be a limitation to our study, but the literature with the term DDH is more recent.

## 5. Conclusions

The incidence of DDH presents fluctuations, not only among European countries, but also between different regions within the same country. Despite certain limitations in the existing body of literature, recent studies have offered valuable data regarding DDH screening. To establish an evidence-based approach for screening at the most appropriate time, a deeper understanding of the natural progression of hip instability and dysplasia, including spontaneous resolution, is necessary. Given the rarity of DDH, it is essential to conduct multicenter studies that evaluate interventions and measure functional outcomes in a standardized manner. It would be beneficial to conduct studies specifically aiming to identify reliable radiologic markers that accurately predict functional outcomes. In any case, the sensibilization of obstetric care specialists is essential. Identifying pre- and perinatal risk factors for DDH and referral to a multidisciplinary team comprised of a neonatologist and a pediatric orthopedic surgeon can result in an early diagnosis, which is crucial for the management of a potential disorder and for the provision of relevant care and follow-up.
